# Effects of an Individually Tailored Web-Based Chronic Pain Management Program on Pain Severity, Psychological Health, and Functioning

**DOI:** 10.2196/jmir.2296

**Published:** 2013-09-25

**Authors:** Dana C Nevedal, Chun Wang, Lindsay Oberleitner, Steven Schwartz, Amy M Williams

**Affiliations:** ^1^VA Connecticut Healthcare SystemDepartment of Clinical Health PsychologyWest Haven, CTUnited States; ^2^Yale University School of MedicineDepartment of PsychiatryNew Haven, CTUnited States; ^3^Wellness & Prevention, Inc.Ann Arbor, MIUnited States; ^4^Social WellthLas Vegas, NVUnited States; ^5^Wayne State UniversityDepartment of PsychologyDetroit, MIUnited States

**Keywords:** Web-based, Internet, chronic pain, treatment outcome, pain management, cognitive behavior therapy, psychology

## Abstract

**Background:**

It is estimated that 30% of adults in the United States experience daily chronic pain. This results in a significant burden on the health care system, in particular primary care, and on the workplace. Chronic pain management with cognitive-behavioral psychological treatment is effective in reducing pain intensity and interference, health-related quality of life, mood, and return to work. However, the population of individuals with chronic pain far exceeds the population of therapists that can provide this care face-to-face. The use of tailored, Web-based interventions for the management of chronic pain could address limitations to access by virtue of its unlimited scalability.

**Objective:**

To examine the effects of a tailored Web-based chronic pain management program on subjective pain, activity and work interference, quality of life and health, and stress.

**Methods:**

Eligible participants accessed the online pain management program and informed consent via participating employer or health care benefit systems; program participants who completed baseline, 1-, and 6-month assessments were included in the study. Of the 645 participants, the mean age was 56.16 years (SD 12.83), most were female (447/645, 69.3%), and white (505/641, 78.8%). Frequent pain complaints were joint (249/645, 38.6%), back (218/645, 33.8%), and osteoarthritis (174/654, 27.0%). The online pain management program used evidence-based theories of cognitive behavioral intervention, motivational enhancement, and health behavior change to address self-management, coping, medical adherence, social support, comorbidities, and productivity. The program content was individually tailored on several relevant participant variables.

**Results:**

Both pain intensity (mean 5.30, SD 2.46), and unpleasantness (mean 5.43, SD 2.52) decreased significantly from baseline to 1-month (mean 4.16, SD 2.69 and mean 4.24, 2.81, respectively) and 6-month (mean 3.78, SD 2.79 and mean 3.78, SD 2.79, respectively) assessments (*P*<.001). The magnitude of the 6-month effects were large. Trends for decreases in pain interference (36.8% reported moderate or enormous interference) reached significance at 6 months (28.9%, *P*<.001). The percentage of the sample reporting fair or poor quality of life decreased significantly from 20.6% at baseline to 16.5% at 6 months (*P*=.006).

**Conclusions:**

Results suggest that the tailored online chronic pain management program showed promising effects on pain at 1 and 6 months posttreatment and quality of life at 6 months posttreatment in this naturalistic study. Further research is warranted to determine the significance and magnitude of the intervention’s effects in a randomized controlled trial.

## Introduction

### Background

Recent estimates indicate that 30% of adults in the United States, or over 93 million people, experience chronic pain each day [1] resulting in significant burden to our health care delivery system [2]. In the United Kingdom, it has been reported that more than 80% of all physician visits were pain-related [3,4]. Chronic pain is also a leading cause of disability and diminished job performance; it is estimated that US businesses lose $61.2 billion per year because of employee pain-related productivity impairment [5]. Treatments for chronic noncancer pain are numerous, often costly, and often associated with a variety of health risks [6-20]. Chronic pain treatments include, but are not limited to, the use of opioid and other analgesic medications [9-11,16,21,22], surgical procedures [19,23], injections [24-26], nerve ablations [15], spinal cord stimulators [20,27], physical and occupational therapies [28], biofeedback [29,30], transcutaneous electrical nerve stimulation (TENS) units [31], psychological interventions [32-41], and comprehensive multidisciplinary pain programs [42-45]. Chronic pain is a complex family of disorders with a wide range of causes and courses (eg, disease, injury), and is frequently maintained or exacerbated by additional psychological, behavioral, social, and environmental factors (eg, stress, depression, sleep dysfunction, physical inactivity, financial stress, legal disability) [14,46-48].

Multiple treatment guidelines exist for chronic pain management [6,8,10,14,18,21-23,27,32,35,43,45,49-53]. Recommended treatments vary by type of pain condition, and many include psychological assessment and interventions as part of a comprehensive treatment strategy [32,35]. Empirical evidence supports the efficacy of a cognitive behavioral approach to chronic pain management on pain intensity and interference, health-related quality of life, mood, and return to work [36,40] delivered face-to-face in individual and group formats [32,54]. Research has shown that offering traditional face-to-face cognitive behavioral therapy (CBT) modules tailored to client needs is an effective approach to chronic pain treatment [34], and allows for a more time-limited and cost-effective approach than treating individual clients using nontailored interventions. However, these delivery formats cannot scale to meet the demand. Many individuals lack local access to cognitive behavioral pain management services delivered by a qualified behavioral medicine specialist [55,56]. Furthermore, among those who have geographic access, cost can be a limiting factor [57], and disparity research has shown that racial and ethnic minorities receive adequate treatment of pain even less often than white patients do [58].

To illustrate the magnitude of this problem, the following thought experiment is provided: It is known that approximately 93 million adults in the United States suffer from chronic pain [1]. In order to provide each person with 8 CBT sessions of 50 minutes for pain management per year, a total of 425,143 behavioral medicine pain management specialists (who carried a heavy caseload of 35 pain patients per week and worked 50 weeks per year) would be needed to provide their care. This is more than 4.5 times the total number of practicing psychologists in the United States [59,60]. Furthermore, if these providers billed at an average rate of just $75 per hour, the total costs associated with this individual face-to-face behavioral pain management care would exceed US $55 billion annually. Clearly, current health care resources, in terms of the number of behavioral medicine specialists and pain management funding dollars, are ill-prepared to meet the demand for chronic pain management.

### Evidence for Internet-Based Treatment

Given that nearly 24 million adults are already seeking help for chronic pain online [61,62], Web-based interventions for chronic pain management offer several distinct advantages over more traditional methods of intervention. First, they can inexpensively scale to provide services to large, diverse populations. Next, they are conveniently accessible around-the-clock from any location with a computer and Internet access. This eliminates many barriers for those living in rural areas, those who lack transportation or have mobility problems, those who must follow nonstandard schedules (eg, shift workers), and those whose child- or elder-care responsibilities limit access to care. Third, the technology has advanced sufficiently to allow for a user experience that is deeply tailored and personalized to the individual’s unique symptoms, circumstances, needs, issues, barriers to change, etc. Tailored messaging has been effectively used with a variety of health conditions (eg, diabetes, obesity, hypertension, heart disease) and in a variety of contexts (eg, Internet website, interactive voice response, mobile text messages) for both health promotion and condition management [63-68], although application to chronic pain has been rare to date. Tailoring is accomplished most efficiently through online algorithm-based approaches that quickly and accurately assess a large number of factors to produce content tailored at the sentence fragment level and matched to the client’s needs and motivations. Fourth, within the context of personalized content, the intervention can be delivered with nearly perfect consistency based on sound psychobehavioral theory and established guidelines. Fifth, the privacy afforded by Web-based interventions may be particularly appealing to individuals who avoid face-to-face psychological interventions because of stigma concerns. Finally, Internet-based treatments can be employed as part of the continuum of care that ranges from stand-alone care to adjunct care for those receiving other therapies, including, but not limited to, pharmacotherapy, physical therapy, or more traditional psychobehavioral interventions. Internet-based interventions may also prove to be cost-effective first-line interventions in stepped-care programs, or useful as booster sessions in the weeks and/or months following traditional face-to-face interventions.

Research has shown that Web-based treatment methods can be beneficial in managing chronic health conditions and promoting health [63,69-73]. A recent meta-analysis quantified the salubrious effects of computer-delivered interventions for health promotion [71]. Web-based interventions for chronic back pain [69], chronic headaches [74], pediatric pain [75], and pain in older adults [76] have also shown promise. The meta-analytic review by Macea et al [77] concluded that randomized controlled studies of Web-based cognitive behavioral pain management result in small but consistent reductions in subjective pain. However, few of these studies used highly tailored interactive programming [55], and some included telephone or face-to-face components [77]. Given the high prevalence of chronic pain, and pervasive costs in terms of diminished quality of life, elevated health care usage, diminished productivity, combined with the inadequate supply of appropriately trained behavioral health specialists, we believe that there is a clear need to develop and test promising new methods for delivering psychobehavioral interventions for chronic pain conditions in innovative ways via the Internet. These challenges necessitate that new interventions be clinically effective, scalable, accessible, and financially sustainable.

### Study Goals

The purpose of this study was to examine the naturalistic outcomes of a Web-based chronic pain management program that is structured around well-established chronic pain behavioral treatment guidelines [6,14,70,78] and tailored to each participant’s unique needs. In addition to the overall effectiveness of the program in reducing pain, reductions in interference with daily activities and work, improvement in quality of life and health, and reductions in stress were examined. Furthermore, this study sought to elucidate participant perceptions of program usability and program quality, and whether certain personal characteristics predict maximum benefit of the Web-based chronic pain management program.

## Methods

### Participants and Procedure

Eligible participants were either employed by 1 of 37 participating US companies or a member of 1 of 18 participating US health care plans. Participating employers and health care plans purchased the Web-based, digital pain management program (HealthMedia Inc. *Care for your Pain* digital health-coaching program) as part of their population health offerings and/or health benefit structure. The programs were offered at no additional cost to eligible individuals.

Prospective participants were recruited by mailings, emails, and posted communications about the digital health-coaching programs, including the pain management program. The invitation to participate was sent by the employer or health care plan to all eligible participants. The invitation contained instructions on how to access the digital health-coaching programs and provided an access code necessary for website sign-up.

As part of the online sign-up process, potential participants were instructed to read a statement of informed consent before taking part in the study. Participants could choose to opt-in to the study after they understood the details of participation, potential benefits and risks, and their rights as a participant. The informed consent and study protocol were reviewed and approved by the Allendale Institutional Review Board. After consent and enrollment, participants were directed to all available digital health-coaching programs. Programs were entirely automated via tailoring algorithms and did not include telephone, email, or face-to-face contact with another person. Some participants received a tailored email message suggesting specific programs on the basis of their self-reported symptoms; others selected programs from a menu of all available programs. All participants who self-selected the pain management program (regardless of method) received this message at the beginning of the program: “Before we get started, let’s make sure you’re in the right place. This program isn’t intended for people suffering from the following: acute pain, cancer pain, pelvic or abdominal pain. If you are experiencing these types of pain, contact your health care provider.” Those who continued their entry in this study were invited to complete an online baseline questionnaire that included 58 questions querying demographics, medical and psychological conditions, pain, general well-being, and daily functioning. This information was used to tailor program content and as a baseline measure of self-reported outcomes. Only very brief measures of each construct could be included to attenuate participant burden in this study, which did not compensate participants for the time they spent filling out assessment questionnaires.

All participants who entered the pain management program and completed the 1-month and 6-month follow-up evaluations were included in analyses, and only the first enrollment into the pain program was used for analysis (some participants enrolled in the program multiple times). There were 645 unique participants who provided informed consent and participated in the program between October 10, 2007 and September 15, 2011. Participants did not receive any compensation for participating in this study.

### Pain Management Online Program

The Web-based pain management digital coaching program is a commercially available (albeit not direct to consumers) behaviorally oriented program that uses proprietary technology to tailor the end-user experience such that it emulates an interaction with a behavioral pain management expert. The program uses patient self-report data and algorithms developed by expert clinicians to provide differing information and interventions based upon each participant’s unique pattern of responses to an interactive consultation that queries the end user regarding type, quality, and location of pain, mood, stress, sleep, current methods of managing pain, level of motivation, and self-efficacy to manage pain, and use/nonuse of prescription pain medications.

The online pain management program integrates evidence-based theories of cognitive behavioral treatment [39,79-85], chronic disease self-management [86], motivational enhancement [87], and theories of health behavior change, including social cognitive theory, theory of reasoned action, theory of planned behavior, and self-determination theory [88-95].

**Figure 1:**
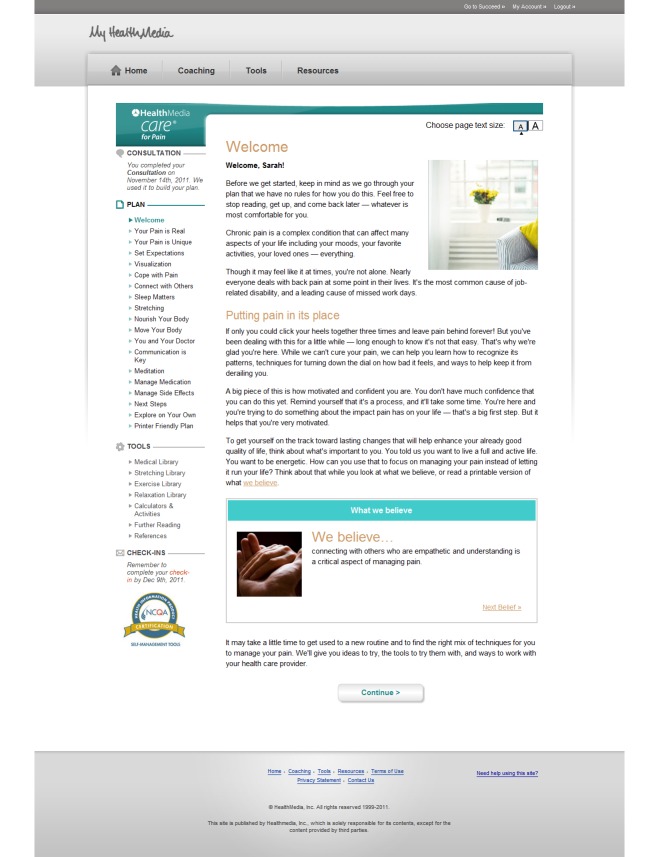
Welcome page of the Web-based pain management program.

**Figure 2:**
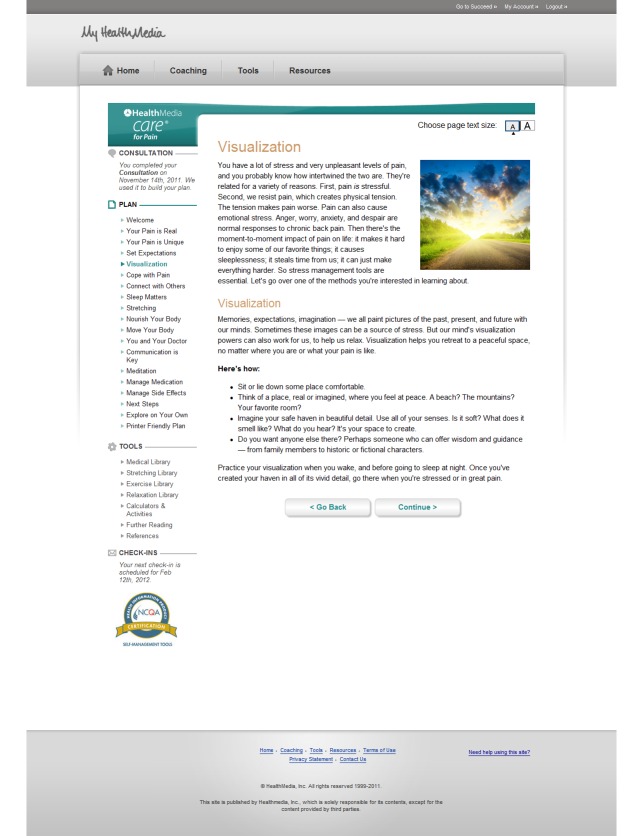
Visualization page of the Web-based pain management program.

The program provides ongoing feedback, usually 1 to 4 sentences long, between pages of the questionnaire to emulate a live coaching experience. Immediately after completing the baseline questionnaire, participants were provided with a tailored personal action plan and access to online pain management tools and library. The personal action plan consisted of 18 tailored Web pages. Regardless of the specific content given to a particular user, each user was provided with a welcome page (see Figure 1) and the following content: setting expectations, managing stress, coping with pain, accessing social support, healthy sleep, nutrition, exercise, improving doctor–patient relationships, medication adherence, and chronic disease self-management. The length of the individual pages varied depending on the particular needs of the user (see Figure 2). Appointment and medication reminders and pain, medication, activity, and sleep logs were included among the tools. The library included psychoeducational materials on self-management, coping, stress management, medication adherence, nutrition, exercise, relaxation, pain conditions, cognitions, social support, comorbid concerns (eg, sleep, stress, mood), doctor–patient relationship and communication, and activity impairment at work and home.

**Figure 3:**
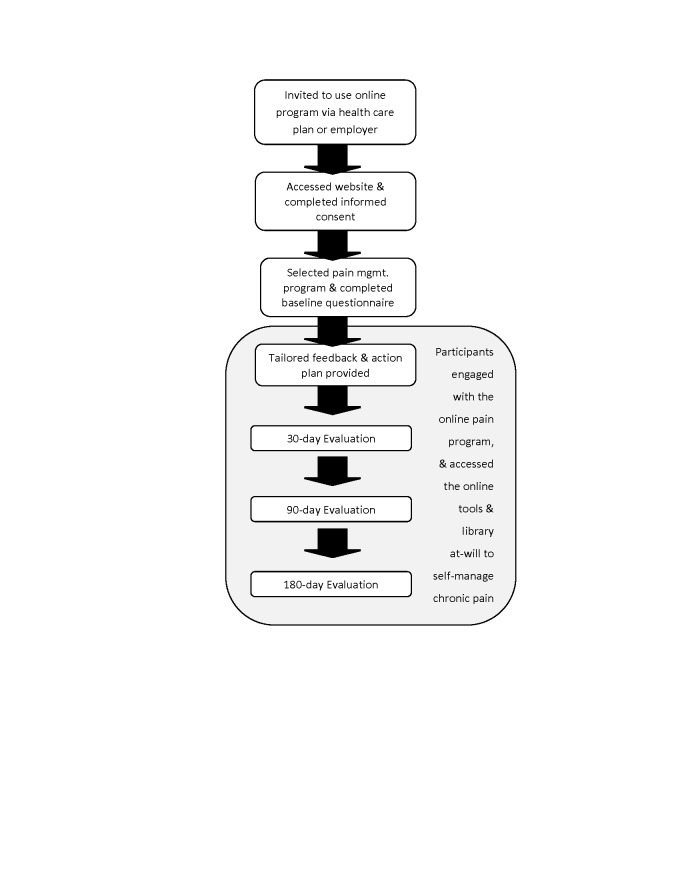
Program architecture and study design.

Program content was delivered through text, images, videos, and interactive tools. During the study period, participants engaged with the program at-will via unlimited access to the action plan, online tools, and library to self-manage their chronic pain. Participants were invited by email to complete follow-up assessments 1 and 6 months after enrolling in the program. The program architecture and study design is displayed in Figure 3.

### Measures

#### Pain Outcomes

Pain is commonly assessed in multiple domains including subjective pain intensity and unpleasantness ratings, interference with daily life, and ability to manage pain. We assessed each of these domains of pain experience. Pain intensity and unpleasantness over the past week were measured using a standard, well-validated 0-10 numeric rating scale with anchors at 0 (no pain or no unpleasantness) and 10 (extreme pain or extreme unpleasantness) [96,97]. To assess the impact of pain on daily activities, we assessed current interference of pain in everyday life using a single item based on a commonly used measure [98]. Pain interference was rated by participants as none (eg, no pain impact on activities), mild (eg, reduced productivity at work or at home), moderate (eg, frequent absences from work, inability to care for family, or inability to do leisure activities), or enormous (eg, inability to work, inability to care for myself, difficulty sleeping, or difficulty walking).

Current level of motivation and confidence to manage pain were also measured using a 0-10 numeric rating scale with anchors at 0 (not at all motivated or not at all confident) and 10 (very motivated or very confident).

#### General Functioning Outcomes

Chronic conditions are frequently associated with lower life and health quality ratings. In this study, quality of health was measured using 1 item from the Centers for Disease Control and Prevention Health-Related Quality of Life 4-Item Measure (CDC HRQOL-4) [90]; quality of life was measured using an adapted version of the same item. The items “Would you say that in general your health is...” and “Would you say that in general your quality of life is...” were provided with response options of poor, fair, good, very good, or excellent.

Stress was measured at each time point with a single item: “How much stress do you feel in a typical day?” Responses were provided on a 4-point scale anchored with the following descriptions: none, not much, some, and a lot.

Depression symptoms were measured at baseline using the 10-item Center for Epidemiologic Studies Depression Scale (CES-D), Boston Form [99]. We used the established cutoff value of 4 or more to indicate a positive depression screen [100].

### Statistical Analyses

Statistical analyses were conducted using SPSS for Windows version 18.0 (SPSS Inc, Chicago, IL, USA). General linear model repeated measures (for continuous variables) or Cochran Q tests (for binary variables) were used to examine baseline, 1-, and 6-month outcomes. Post hoc pairwise tests were conducted by paired sample *t* tests (for continuous variables) or McNemar tests (for binary variables). Independent sample *t* tests (for continuous variables) or chi-square tests (for categorical variables) were applied to examine the between-group differences on baseline data of (1) participants who improved at 1 or 6 months on pain ratings with participants who did not, and (2) participants who completed the 1- and 6-month evaluation with participants who did not. Any *P* value <.05 was considered statistically significant, unless otherwise noted. Each of the pairwise comparisons was tested at a significance level of .017 (.05/3). Effect sizes were reported, when applicable, using Cohen's *d*, and odds ratio (95% CI).

## Results

### Sample Description

Descriptive characteristics of the sample are provided here to aid in the interpretation of the data (eg, extent of generalizability). Participants resided in 38 of the 50 United States. The average participant age was 56 years (SD 12.83). Data on marital status and education level were not collected in this study. At baseline, participants had an average pain intensity rating of 5.30 out of 10 (SD 2.46), and an average unpleasantness rating of 5.43 out of 10 (SD 2.52) in which higher numbers reflect greater pain. Participants were motivated to manage their pain (mean 8.58, SD 1.80), and moderately confident that they could manage their pain (mean 6.94, SD 2.50) on the same 0-10 scale (see Table 1).

At study enrollment, 82.6% (285/345) of participants reported taking medication to relieve their pain, and 54.5% (351/644) of participants used medication every day or nearly every day in the past month. At baseline, participants had an average depression score of 2.70 (SD 2.45) out of a possible 10 points on the CES-D Boston Form [79]. Information on self-reported pain treatments and treatment effectiveness is reported in Table 2.

### Evaluation Submitter and Nonsubmitter Comparisons

We compared program participants who completed the 1- and 6-month evaluations with participants who did not submit evaluations. Evaluation submitters and nonsubmitters did not differ in baseline demographic characteristics (ie, gender, ethnicity), duration of pain condition, quality of life and quality of health ratings, stress level, depression symptoms, the interference of pain in work and life, level of social support for pain, pain medication adherence, health care provider visits, emergency room visits, hospital admissions, or participation in psychological counseling or therapy for pain. Baseline pain intensity and pain unpleasantness ratings also did not differ between groups.

Some other differences between evaluation submitters and nonsubmitters were identified. Evaluation submitters were, on average, nearly 7 years older than nonsubmitters (mean age 56.2 vs 49.4, *P*<.001, *d*=0.53). Evaluation submitters were also less likely to work outside the home (*P*<.001, OR 0.54, 95% CI 0.45-0.63). Evaluation submitters were more motivated to manage their pain at baseline, (*P*<.001, *d*=0.21) and more confident that they could do so (*P*=.001, *d*=0.13). Evaluation submitters also rated their overall quality of sleep higher than noncompleters (*P*<.001, *d*=0.23). Finally, evaluation submitters were more likely than nonsubmitters to see their primary care physician (*P*=.02, OR 1.23, 95% CI 1.04-1.46), rheumatologist (*P*=.01, OR 1.40, 95% CI 1.07-1.82), or physical therapist (*P*=.02, OR 1.31, 95% CI 1.05-1.63) for pain, but less likely to see a chiropractor (*P*=.004, OR 0.69, 95% CI 0.53-0.89) for pain management. They were also more likely to take medication daily to relieve pain (*P*=.02, OR 1.21, 95% CI 1.03-1.41).

### Program Usability

Participants indicated their satisfaction with and their perceptions of the usability of the online pain management program. At 1-month postenrollment, 82.6% (523/633) rated the program good or better given choices of poor, fair, good, very good, and excellent. Furthermore, most participants gave the program high usability ratings. Specifically, on a 5-point scale ranging from disagree (=1) to agree (=5), the percentage of participants who rated the program a 3 or better on the following items are as follows: 95.3% (602/632) read the information completely; 96.8% (609/629) found the information easy to read; 88.6% (551/622) found the information to be personally relevant; 93.8% (590/629) found the program easy to use; 90.5% (564/623) found the interactive exercises to be more helpful than simply reading a book or an article online; and 91.5% (558/610) experienced no technical difficulties while using the product.

### Pain Outcomes

Participants’ pain intensity ratings decreased significantly following the intervention (*F*_2,1272_ = 142.61, *P*<.001). The average baseline pain intensity rating was 5.30 (SD 2.46) out of a possible 10. Mean pain intensity decreased to 4.16 (SD 2.69) at 1 month and 3.72 (SD 2.73) at 6 months. Post hoc pairwise tests indicated a significant reduction in pain intensity at 1 month compared with baseline (moderate-sized effect, *d*=0.69, *P*<.001), and 6 months compared with 1 month (small-sized effect, *d*=0.26, *P*<.001), and 6 months compared with baseline (large-sized effect, *d*=0.88, *P*<.001).

Pain unpleasantness ratings also decreased significantly following the intervention (*F*_2,1266_ = 143.66, *P*<.001). The average pain unpleasantness ratings were 5.43 (SD 2.52) at baseline, 4.24 at 1 month (SD 2.81), and 3.78 at 6 months (SD 2.79). Post hoc pairwise tests indicated a significant reduction at 1 month compared with baseline (moderate-sized effect, *d*=0.70, *P*<.001), 6 months compared with 1 month (small-sized effect, *d*=0.26, *P*<.001), and 6 months compared with baseline (large-sized effect, *d*=0.90, *P*<.001).

Pain interference was rated by participants as none, mild, moderate, or enormous. At baseline, 36.8% (231/627) of participants reported that they had moderate or enormous degrees of interference in daily life secondary to their pain. The proportion of participants reporting moderate or enormous pain interference decreased significantly following the intervention (1 month: 34.0%, 213/627; 6 months: 28.9%, 181/ 627; Q_2_,_627_= 21.14, *P*<.001). Post hoc pairwise tests indicate a significant reduction at 6 months compared to 1 month (*P*=.004), and 6 months compared to baseline (*P*<.001).

At baseline, participants reported that they were quite motivated to manage their pain (mean 8.58, SD 1.80). Motivation to manage pain did not change significantly following the intervention (1 month: mean 8.39, SD 2.13; 6 month: mean 8.44, SD 2.18; *F*_2,1234_ = 2.79, *P*=.06).

At baseline, participants reported that they were fairly confident that they could manage their pain (mean 6.94, SD 2.50). Confidence in managing pain did not change significantly following the intervention (1 month: mean 7.15, SD 2.60; 6 month: mean 7.11, SD 2.71; *F*_2,1242_ = 2.26, *P*=.11).

### General Functioning Outcomes

Participants rated their quality of life as corresponding to 1 of the following categories: poor, fair, good, very good, and excellent. One-fifth (20.6%, 129/625) of participants rated their quality of life as poor or fair at baseline. The proportion of participants rating their quality of life as fair or poor decreased following the intervention (Q_2_,_625_= 8.00, *P*=.02); 18.1% at 1 month (113/625), and 16.5% (103 /625) at 6 months. Post hoc pairwise tests indicated a significant reduction at 6 months compared with baseline (*P*=.006).

Quality of health was rated using the same categories as quality of life. At baseline, 19.7% of participants reported their quality of health was fair or poor (123/624). This did not change significantly following the intervention (1 month: 16.8%, 105/624; 6 months: 17.1%, 107/624, Q_2_,_624_ = 5.26, *P*=.07).

Participants rated their stress by selecting 1 of the following descriptors: none, not much, some, or a lot. At baseline, 18.9% of participants reported a lot of stress (63/334). This proportion decreased nonsignificantly over time (1 month: 16.2%, 54/334; 6 months: 16.8%, 56/334; Q_2_,_334_= 1.84, *P*=.40).

### Effects of Participation in Additional Online Wellness Programs

We tested to see if participation in additional online wellness programs beyond the pain program resulted in differential program outcomes. None of the group (pain program only: n=256; pain plus additional program or programs: n=389) by time effects were significant (all *P*>.05).

### Treatment Responder Characteristics

Pain intensity and pain unpleasantness were considered primary outcomes in this study. Each participant’s pain intensity and pain unpleasantness ratings were summed and divided by 2 to compute an average pain composite at baseline, 1, and 6 months. Because reductions in pain equal to or greater than 30% are generally accepted as clinically meaningful, participants whose average pain composite score at 1 or 6 months was at least 30% less than their average pain composite score at baseline were defined as treatment responders for that time point [101,102]. Those whose average pain composite did not decrease by at least 30% at 1 or 6 months were defined as nonresponders for that time point. Changes in other measures (eg, stress), although important, were not included in the responder analysis because only a subset of participants reported problems in each secondary outcome at baseline. Among those who submitted follow-up evaluations, treatment response rates were 43.7% (270/618) and 52.4% (324/618) at 1 and 6 months, respectively.

Treatment responders and nonresponders did not differ in baseline demographic characteristics (ie, gender, age, ethnicity, and people living in their household), pain duration, motivation to manage pain, type of health care providers treating their pain, pain medication adherence, or level of social support for pain. Characteristics that distinguished treatment responders from nonresponders are reported in Table 3.

**Table 1:** Baseline sample description (N=645).

Characteristic		%	n	N^a^
Female		69.3	447	645
**Age group (years)**				645
	22-29	2.2	14	
	30-39	8.4	54	
	40-49	17.2	111	
	50-59	33.5	216	
	60-69	23.1	149	
	70-91	15.7	101	
**Race/ethnicity**				641
	White	78.8	505	
	African American	8.4	54	
	Hispanic	5.9	38	
	Other	6.9	44	
**Geographic region of residence**				638
	Northeast	9.4	60	
	South/southeast	17.4	111	
	Midwest	23.7	151	
	West	49.5	316	
**Occupation**				632
	Professional	18.7	118	
	Clerical/admin support	21.7	137	
	Not working outside home	31.5	199	
	Sales/tech support/service	15.7	99	
	Executive/senior manager/administration	5.9	37	
	Production/operator/laborer	6.6	42	
**Pain duration**				253
	<6 months	9.1	23	
	6 months-1 year	9.9	25	
	1-5 years	32.4	82	
	5-10 years	20.6	52	
	>10 years	28.1	71	
**Pain complaints**				645
	Joint	38.6	249	
	Back	33.8	218	
	Osteoarthritis	27.0	174	
	Migraine	15.8	102	
	Neuropathy	15.8	102	
Positive depression screen		33.9	191	

^a^Because of changes to the program questionnaire over time, some items were not asked of all participants (eg, pain duration). The N reported indicates number of participants who responded to the item.

**Table 2:** Baseline self-reported pain treatments and pain management effectiveness (N=645).

Pain treatment		%	n	N
**Pain medication**				643
	No medication	17.4	112	
	Over-the-counter only	29.4	189	
	Prescription only	22.4	144	
	Prescription and over-the-counter	30.8	198	
**Psychological counseling in the past month**				623
	None	92.3	575	
	1-2 times	5.0	31	
	3-15 times	2.7	17	
**Health care providers treating their pain**				645
	Primary care doctor	70.1	452	
	Orthopedic surgeon	14.1	91	
	Rheumatologist	9.6	62	
	Neurologist	8.7	56	
	Chiropractor	10.1	65	
	Physical therapist	14.4	93	
	Pain specialist or anesthesiologist	5.1	33	
	Other	16.1	104	
**Pain treatment effectiveness**				625
	It takes away little or none of pain	17.3	108	
	It takes away some of pain	73.3	458	
	It takes away pain completely	9.4	59	

**Table 3:** Baseline characteristics distinguishing treatment responders from treatment nonresponders at 1 month and 6 months (N=645).

Characteristic	1-Month follow-up^a^			6-Month follow-up^a^		
	OR (95% CI)	Cohen’s *d*	*P*	OR (95% CI)	Cohen’s *d*	*P*
Pain intensity rating		–0.21	.008		–0.22	.005
Pain unpleasantness rating		–0.25	.002		–0.25	.002
Self-report back pain	0.58 (0.41, 0.82)		.002	0.42 (0.30, 0.59)		<.001
Self-report fibromyalgia	0.44 (0.25, 0.75)		.002	0.46 (0.28, 0.76)		.002
Self-report neuropathy	0.46 (0.29, 0.73)		.001	0.51 (0.33, 0.79)		.002
Self-report obesity	0.55 (0.31, 0.97)		.04	0.34 (0.19, 0.61)		<.001
Back is most painful site	0.56 (0.41, 0.78)		<.001	0.53 (0.38, 0.73)		<.001
Better overall sleep quality		0.25	.008		0.21	.02
Screen positive for depression	0.48 (0.33, 0.69)		<.001	0.47 (0.33, 0.67)		<.001
Pain causes anxiety/irritability/depression		–0.36	<.001		–0.39	<.001
Physical activity restricted by HCP	0.59 (0.41, 0.86)		.006	0.58 (0.40, 0.83)		.003
Interference of pain in work and life		–0.36	<.001		–0.33	<.001
Sick days		–0.33	.001		–0.24	.05
Involved in pain-related litigation	0.60 (0.25, 1.40)		.23	0.34 (0.14, 0.82)		.01
Receiving disability compensation	0.40 (0.16, 1.01)		.05	0.34 (0.14, 0.82)		.01
Believe worsening pain indicates one’s condition is worsening	0.49 (0.34, 0.72)		<.001	0.69 (0.48, 0.99)		.04
Believe pain is just a normal part of life/aging		0.31	<.001		0.18	.03
Refuse to let pain stop me from doing what i enjoy	1.44 (1.02, 2.01)		.04	1.11 (0.80, 1.55)		.52
Importance of living a full and active life		0.30	.01		0.26	.03
Importance of working to earn a living		0.17	.16		0.26	.04
Importance of being responsible for my health		0.21	.08		0.24	.04
Importance of limiting health care costs		0.06	.61		0.26	.03
Take prescription medication	0.52 (0.38, 0.72)		<.001	0.58 (0.42, 0.81)		.001
Take prescribed opioid medication	0.36 (0.24, 0.52)		<.001	0.43 (0.30, 0.62)		<.001
Have been taking medication “too long”	0.49 (0.24, 0.99)		.04	0.43 (0.22, 0.86)		.01
Believe medication is not working	0.53 (0.27, 1.07)		.07	0.47 (0.24, 0.91)		.02
Number of doctor visits		–0.25	.001		–0.12	.14
Participate in counseling or psychotherapy		–0.58	.006		0.14	.65

^a^Positive *d* values and OR <1 indicate that treatment responders endorsed the variable more often or scored higher on this variable than nonresponders. Negative *d* values and OR >1 indicate that treatment responders endorsed the variable less often or scored lower on the variable than nonresponders.

## Discussion

### Principal Findings

In this study, we examined the longitudinal effects of a tailored Web-based chronic pain management program on multiple dimensions of pain experience (ie, intensity, unpleasantness, and interference), motivation to manage pain, confidence in ability to manage pain, quality of life and health, and stress among US adult employees and health care plan members who self-reported chronic pain and completed 1- and 6-month follow-up evaluations. Our main findings suggest that the tailored online chronic pain management program exerts significant beneficial effects on pain intensity, pain unpleasantness, pain interference, and quality of life 6 months after program enrollment. The effects on pain intensity and unpleasantness were notably large in magnitude. Average motivation to manage pain was initially quite high (8.58/10), and did not significantly change during follow-up. Confidence in managing pain did not change significantly over the course of the study, but remained at a consistently moderate level (approximately 6.5/10). The results also suggest that most participants found the program to be user friendly and of good or better quality. Overall, our results indicate that this tailored, online, chronic pain management program is a promising, low-cost, user-friendly intervention with the advantage of virtually unlimited scalability. However, future randomized controlled studies are necessary to ensure that the promising results of this study were because of the effects of the intervention and not the simple effects of time.

Next, we conducted tests to determine what characteristics distinguished the participants whose pain responded favorably to the intervention from those who did not experience improvement in their pain. First, we found that lower pain intensity and unpleasantness were associated with treatment response, which suggests that the intervention may be most helpful for people experiencing mild to moderate pain. Other characteristics predictive of treatment response include better baseline sleep quality, better emotional functioning, more adaptive cognitions, and a goal-oriented cognitive style. These findings suggest that treatment responders have somewhat better psychological health before treatment. This may indicate that the program works best for people with some psychological resilience. Alternatively, these findings may indicate that the program does not address co-occurring problems of mood, anxiety, stress, emotion regulation, or sleep adequately, leading individuals with these problems to benefit less from treatment. Thus, the effectiveness of the online pain management program might be improved by increasing tailoring and content to address these potential shortcomings. Similarly, treatment responders appeared to be physically healthier (fewer fibromyalgia, spinal, or neuropathic complaints, less obesity and chronic disease, fewer activity limitations), use less medications, and use fewer health services. Overall, this pattern suggests that the Web-based chronic pain management intervention may be most effective for those individuals with mild or moderate chronic pain that have better overall psychological and physical health at the time they initiate the program. Individuals with more numerous comorbidities or certain pain conditions (eg, spinal, neuropathic, or fibromyalgia pain) may require a more intensive, disease-specific, face-to-face intervention to achieve optimal outcomes. Alternatively, Web-based pain management interventions could benefit from program development to tailor the program content more deeply to address the needs of these individuals.

### Broader Implications

Ease of access may be especially important for people with chronic pain when pain flares unexpectedly and immediate access to resources that promote adaptive pain coping strategies (eg, relaxation audio clips, imagery, distracting techniques) can provide relief. Additionally, the convenience of using Internet-based pain management from home can lead to greater access to the program tools, reaching individuals with pain that might not otherwise seek out treatment because of disability, physical limitations, lack of transportation, distance, or time [89].

As a result of the easy access to Internet-based pain information, there is a virtually unlimited amount of online information available to individuals experiencing chronic pain. The downside to this is that the sheer amount of available information can be overwhelming, and the validity of this information is often specious. Tailored, online, evidence-based pain management programs identify the unique needs of individual chronic pain clients, match these needs with key information and evidence-based interventions, and deliver them in manageable amounts [37,41,91]. Treatments can be tailored on the varied presentations of chronic pain patients: diagnoses (eg, osteoarthritis, headaches, back pain), personality, motivation to manage pain, comorbid symptoms (eg, depression, insomnia, obesity), preferences of the individual, demographics, and more. As evidence accumulates that tailored online interventions can effectively manage pain and other health conditions, we may find that they are more cost-effective, acceptable, convenient, and sustainable than many traditional clinic-based interventions. Web-based pain management programs may also be cost-effective first-line interventions in stepped-care models of pain management, or targeted toward those healthier patients with less severe manifestations of chronic pain, such as the treatment responders in this study.

### Study Strengths

This study has several noteworthy strengths. First, the participant sample was large and diverse with regard to geographic location, occupation, race/ethnicity, and age. This sample offers greater generalizability of results to typical chronic pain sufferers than results derived from undergraduate psychology student convenience samples that are ubiquitous in the psychology literature. Next, the nature of the online pain management program allows us to conclude that the intervention was implemented with nearly perfect consistency. Third, the naturalistic design and longitudinal follow-up support the external validity and generalizability of the study in comparison with tightly controlled laboratory-based studies. Finally, we reported effect sizes and odds ratios to enable the reader to readily assess the clinical significance/magnitude of change associated with our outcomes, and easily compare these with other pain management study results.

### Study Limitations

This study also has some limitations that should be made explicit. First, because this was an uncontrolled study, we cannot be certain that the findings were not the result of regression to the mean, or effects of time rather than the intervention itself. Thus, these results should be interpreted as promising evidence, but certainly not conclusive. Second, because participants in this study self-selected the online pain management program, we cannot determine if the pattern of effects we observed generalize to others who would not elect to participate in this program. However, given that pain patients nearly always self-select their own pain management methods from available options (eg, medications, physical therapy, chiropractic manipulation, surgery, rest), this design has ecological validity. Third, limitations in the available data preclude examination of the extent to which participants interacted with the program. Although the program is designed to be self-paced and accessed at-will by the individual, it would have been desirable to test dose–response relationships. Fourth, all the outcomes were based upon brief self-report data and monomethod measurement can be subject to reporting biases (eg, socially desirable responding). Objective measures of pain disability (eg, functional tests) and/or collateral reports of pain behavior would have addressed this problem, but were not logistically possible. Longer, more commonly used outcome measures with well-established validity evidence would also have improved the quality of this study. However, participant burden was the impetus for selecting very brief measures in this naturalistic study. Because participants were not compensated for the significant time it takes to complete lengthy measures (as is the norm in laboratory-based research) the pragmatic decision was made to design the programs with the very brief measures of outcome.

### Future Directions

First and foremost, well-powered randomized controlled trials of tailored Web-based pain management programs are needed to determine time and self-selection effects versus intervention effects on pain and psychosocial outcomes. Future studies should also make use of well-validated measures of pain outcomes [97] and take necessary steps (eg, participant compensation for time) to encourage completion of measures.

Next, future researchers should also measure change in theoretically relevant proximal variables (eg, pain cognitions, use of exercise/relaxation/visualization for pain management) to test mechanisms of the effects of tailored Web-based pain management programs. Once efficacy is established, there will be a need for dismantling studies to examine whether there are differential effects of tailoring program content on certain variables (eg, gender, stage of change, job type) versus others. Finally, empirical evidence showing which variables exert the greatest tailoring effects (vs those with weak or no effects) on outcomes should guide development of future interventions and improvement of those currently in existence.

## Abbreviations

CBT: cognitive behavioral therapy
TENS: transcutaneous electrical nerve stimulation
